# A novel circular RNA, hsa_circ_0030998 suppresses lung cancer tumorigenesis and Taxol resistance by sponging miR‐558

**DOI:** 10.1002/1878-0261.12852

**Published:** 2021-02-10

**Authors:** Xiaoping Li, Yiling Feng, Bo Yang, Ting Xiao, Haixia Ren, Xi Yu, Lei Li, Mingjiang Li, Weidong Zhang

**Affiliations:** ^1^ Department of Thoracic Surgery Tianjin First Central Hospital China; ^2^ Department of Oncology Armed Police Characteristic Medical Center Tianjin China; ^3^ College of Pharmacy State Key Laboratory of Medicinal Chemical Biology Nankai University Tianjin China; ^4^ Department of Pharmacy Tianjin First Central Hospital China; ^5^ Department of Respiratory Tianjin First Central Hospital China

**Keywords:** hsa_circ_0030998, lung cancer, miR‐558, MMP1, MMP17, Taxol resistance

## Abstract

Circular RNAs (circRNAs) are single‐stranded RNAs which form a covalently closed continuous loop. Although originally shown to be non‐protein‐coding, some circRNAs can give rise to micropeptides. circRNAs have also been shown to play essential regulatory roles in a variety of developmental and disease processes. In a previous study, hsa_circ_0030998 was identified as a circRNA downregulated in lung cancer, but its potential implications and mechanisms in lung cancer were not addressed. Here, we showed that overexpressing circ_0030998 decreased proliferation, migration, and invasion of lung cancer cells, while also dampening resistance to Taxol, a classical antitumor drug. Depleting circ_0030998 reversed these phenotypic effects. A high circ_0030998 expression was correlated with a high survival rate in lung cancer patients. Additionally, we found circ_0030998 could downregulate miR‐558 expression, serving as a microRNA sponge. In conclusion, our data support that hsa_circ_0030998 can slow down the progression of lung cancer by targeting miR‐558 and suppress malignant phenotypes such as proliferation, migration, and invasion progression of lung cancer cells. Therefore, we highlight that circ_0030998 could be a novel tumor suppressor of lung cancer.

## Graphical abstract

In the normal lung tissues, hsa_circ_0030998 get a balance level. Our study verified that the downregulated hsa_circ_0030998 can inhibit proliferation, migration, invasion and Taxol resistance by sponging miR‐558 in lung cancer. Therefore, the hsa_circ_0030998/miR‐558 axis might be a possible diagnostic and therapeutic target in lung cancer patients.

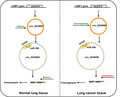

**Abbreviations**
circRNA: circular RNA
IP: immunoprecipitation
LAMP1: lysosomal‐associated membrane protein 1
miRNA: microRNA
MMP: matrix metalloproteinase
NC: negative control

## 1 Introduction

Lung cancer has become the cancer with the highest incidence and mortality among malignant tumors worldwide [1]. The current treatment methods for lung cancer mainly include surgery, chemotherapy, radiotherapy, local treatment, targeted therapy, and immunotherapy [2]. Although with the advancement of treatment technology, the survival rate of lung cancer patients has improved, with the 1‐year survival rate increasing from 34% (1975–1977) to 45% (2008–2011), the prognosis of most patients is still poor [3]. The main causes of poor prognosis and high mortality in cancer patients are recurrence and metastasis [4]. Currently, Taxol, a classical antitumor drug with strong antitumor activity, is the standard first‐line treatment for advanced lung cancer [5, 6]. However, approximately 50–70% of lung cancer patients do not benefit from Taxol treatment in clinical practice [7]. Therefore, metastasis, recurrence, and drug resistance are vital causes of the failure of lung cancer treatment, and research on lung cancer therapy needs to be explored in depth.

Recently, increasing evidence has shown that the development of lung cancer is the result of a series of genetic and molecular changes [8]. Noncoding RNAs (ncRNAs), which account for 98–99% of the human genome, have essential effects in gene expression and regulation [9]. ncRNAs include rRNAs, tRNAs, snRNAs, siRNAs, microRNAs (miRNAs), long noncoding RNAs (lncRNAs), and circular RNAs (circRNAs), which have been studied extensively in recent years [10]. Among these ncRNAs, circRNAs do not have a 5'‐end cap structure and 3'‐end poly (A) tail structure, and the 5'‐end and 3'‐end are covalently linked end to end to form a circular structure [11]. In addition, circRNAs are not easily degraded by nucleic acid exonuclease (RNase R) and can be stably stored in organisms [12]. With the development of RNA sequencing technology, an increasing number of circRNAs have been discovered [13]. At present, circRNAs have become hotspots in tumor research. Multifarious circRNAs have been reported to play essential regulatory roles in a variety of cancer processes [14, 15, 16]. However, the current effects of hsa_circ_0030998 in lung cancer, especially metastasis and drug resistance, have not been reported.

The known mechanisms of circRNAs are as follows. First, circRNAs serve as sponges for microRNAs (miRNAs) and can relieve the inhibition of miRNAs on their downstream target genes, thus regulating the crucial signaling pathways of cancer development [17]. Second, circRNAs, as scaffold proteins, can regulate cell cycle‐related proteins through interactions with proteins [18]. Finally, circRNAs can directly regulate the transcription, splicing, and expression of parental genes [19]. Therefore, it is of vital importance to explore the mechanism of hsa_circ_0030998 in the progression of lung cancer.

In this study, we verified the expression of hsa_circ_0030998 in lung cancer tissues and Taxol‐resistant and Taxol‐sensitive lung cancer cells. We further determined that hsa_circ_0030998 can act as a miR‐558 sponge, thus affecting the proliferation, Taxol resistance, invasion, and metastasis of lung cancer cells. Therefore, we aimed to explore the potential functions and mechanisms of hsa_circ_0030998, which might be involved in the malignant processes of lung cancer, in an attempt to develop new ideas for the diagnosis and treatment of lung cancer.

## 2 Results

### 2.1 hsa_circ_0030998 is downregulated in lung cancer and encoded by LAMP1 exon 3

**Fig. 1:**
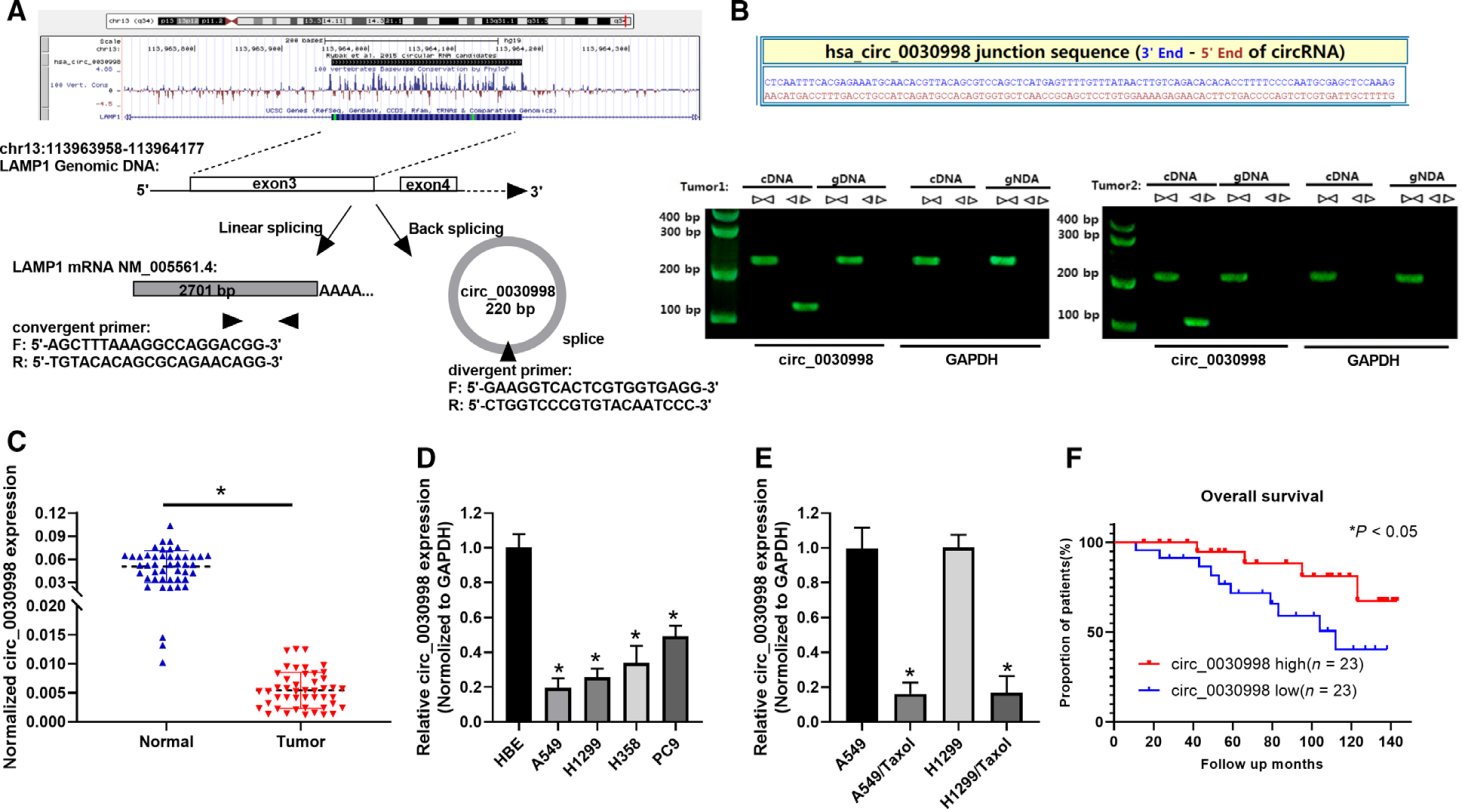
hsa_circ_0030998 has been identified to have low expression in lung cancer. (A) The location of circ_0030998 is indicated on the chromosome, and the formation of circ_0030998 from LAMP1 mRNA (exon 3) is illustrated. (B) The splice junction sequence of circ_0030998 is displayed. PCR assays were conducted to verify the circular structure of circ_0030998. (C) qRT–PCR results of circ_0030998 in lung cancer and nontumor normal tissues are shown. (D) circ_0030998 expression was also determined through qRT–PCR analysis in HBE and lung cancer cell lines (A549, H1299, H358, and PC9). (E) qRT–PCR assay was also applied to monitor circ_0030998 expression in Taxol‐resistant or nonresistant A549 and H1299 cells. (F) The relationship between prognosis and circ_0030998 was analyzed through the Kaplan–Meier in lung cancer. Data represent the mean ± SD from three independent experiments. Student’s t‐test with two biological dependent or independent replicates was used to determine statistical significance;*P < 0.05

hsa_circ_0030998 was indicated as a lung cancer‐downregulated circRNA by our previous study [20]. As exhibited in Fig. 1A, the schematic diagram of the circBase database (http://www.circbase.org/) showed that it is located on chr13:113963957‐113964177 and originated from LAMP1 mRNA (exon 3). To confirm hsa_circ_0030998, we designed convergent and divergent primers based on the junction sequence. Then, we adopted divergent primers to amplify circ_0030998 and convergent primers to amplify LAMP1 (mRNA) by PCR assay using gDNAs (genomic DNAs) and cDNAs as templates. We discovered that circ_0030998 was only amplified using divergent primers in cDNAs (Fig. 1B). In addition, we found that the linear form of LAMP1 was dramatically reduced after RNase R administration, while circ_0030998 resisted RNase R digestion in A549 and H1299 cells (*P* < 0.05, Fig. S1A,B). Simultaneously, we also verified that compared with linear LAMP1, the circular transcript circ_0030998 was not inhibited by actinomycin D in A549 and H1299 cells (*P* < 0.05, Fig. S1C,D). Subsequently, we determined the expression of circ_0030998 in lung cancer and nontumor normal tissues using qRT–PCR analysis. As displayed in Fig, 1C, circ_0030998 expression demonstrated a significant decrease in lung cancer tissues versus nontumor normal tissues (*P* < 0.05). Our data also showed that high expression of circ_0030998 was negatively related to invasion (*P* = 0.030) and distal metastasis (*P* = 0.040) and TNM stage (*P* = 0.019) in lung cancer patients (Table 1). Similarly, our data also revealed that circ_0030998 was markedly downregulated in lung cancer cells (A549, H1299, H358, and PC9) compared with HBE cells (*P* < 0.05, Fig, 1D). Above all, our results revealed that the level of circ_0030998 was prominently reduced in Taxol‐resistant A549 and H1299 cells compared with the Taxol‐sensitive A549 and H1299 cells (*P* < 0.05, Fig, 1E). Additionally, through the Kaplan–Meier plots, we revealed that higher circ_0030998 expression resulted in a higher survival rate of lung cancer patients (*P* < 0.05, Fig, 1F).

**Table 1:** The correlation between hsa_circ_0030998 expression and clinical features of lung cancer

Clinicopathologic Characteristics	No. of patients	hsa_circ_0030998		*P* value
		High	Low	
*Age (year)*				
>60	26	15 (57.7%)	11 (42.3%)	0.558
≤60	20	12 (60.0%)	8 (40.0%)	
*Gender*				
Male	28	17 (60.7%)	11 (39.3%)	0.482
Female	18	10 (55.6%)	8 (44.4%)	
*Tumor size (cm)*				
<3	25	15 (60.0%)	10 (40.0%)	0.194
≥3	21	9 (42.9%)	12 (57.1%)	
*Differentiation grade*				
Well/moderately	24	16 (66.7%)	8 (33.3%)	0.072
Poorly/undifferentiated	22	9 (40.9%)	13 (59.1%)	
*Invasion*				
T0–T2	33	22 (66.7%)	11 (33.3%)	0.030*
T3–T4	13	4 (30.8%)	9 (69.2%)	
*Distal metastasis*				
M0	39	28 (71.8%)	11 (28.2%)	0.040*
M1	7	2 (28.6%)	5 (71.4%)	
*TNM stage*				
I and II	35	24 (68.8%)	11 (31.4%)	0.019*
III and IV	11	3 (27.3%)	8 (72.7%)	

* *P* < 0.05, TNM stage: pathologic tumor, node, metastasis stage.

In addition, we found that the Alu elements of exon 3 on both sides complement each other in opposite directions, and the sequence is shown (Fig , S2A,B). Based on the data from the Kaplan–Meier plotter, we also discovered that the high expression of LAMP1 also displayed a long survival rate of lung cancer patients (Fig. S2C). The correlation analysis also demonstrated that there was a positive correlation between hsa_circ_0030998 expression and LAMP1 expression in lung cancer (*r* = 0.6529, *P* < 0.0001, Fig. S2D). These data suggested that hsa_circ_0030998, which is encoded by LAMP1 exon 3, is downregulated in lung cancer tissues and cells, and its high expression is related to a good prognosis.

### 2.2 Overexpression of circ_0030998 prevented the malignant progression of lung cancer cells

**Fig. 2:**
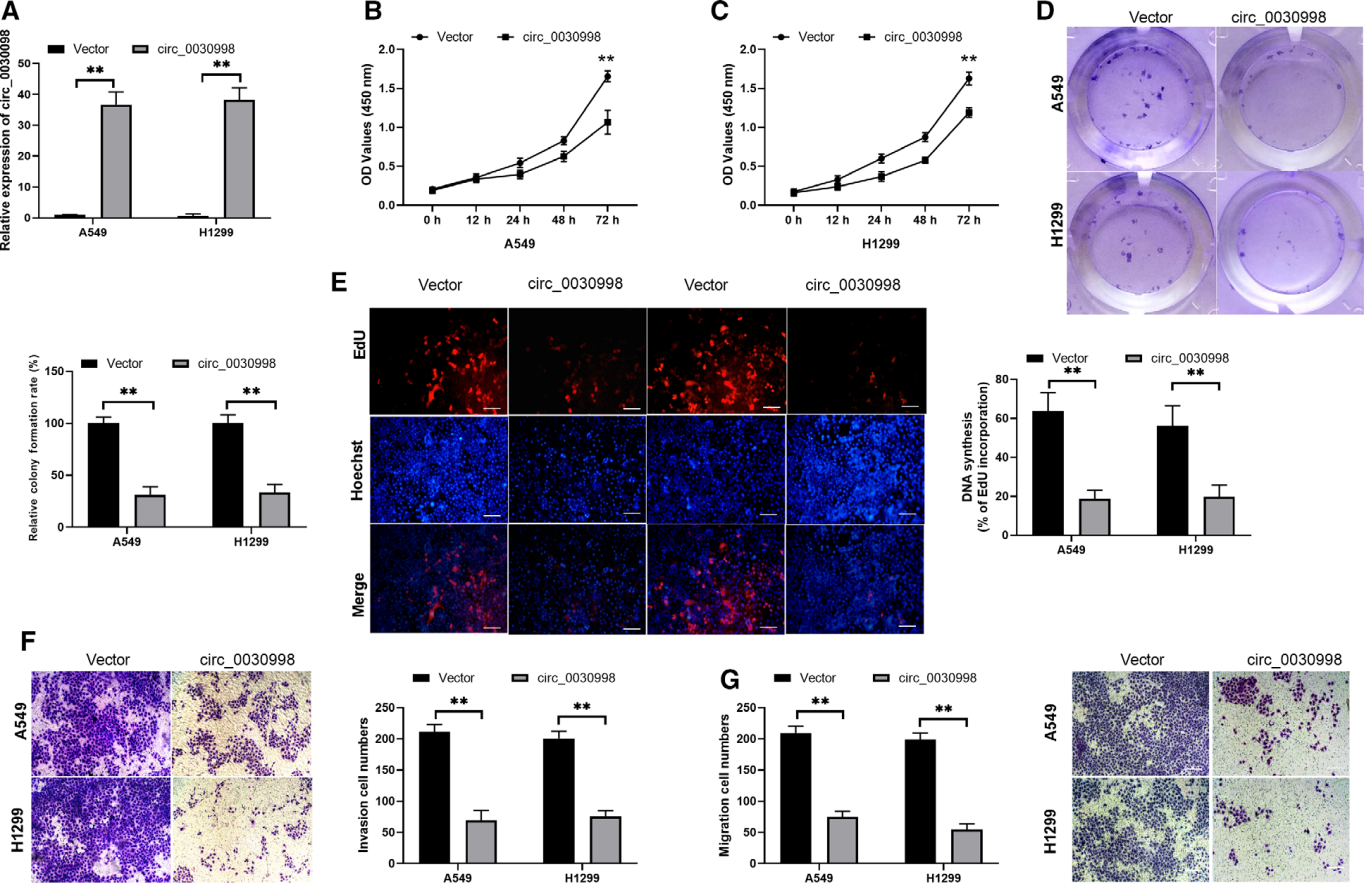
Overexpression of circ_0030998 prevented the malignant progression of lung cancer cells. (A) The overexpression of circ_0030998 in A549 and H1299 cells was identified by applying qRT–PCR assay. (B,C) CCK‐8 assay exhibited the proliferation inhibition of A549 and H1299 cells after circ_0030998 plasmid transfection. (D) Colony formation assays revealed that circ_0030998 overexpression resulted in a decrease in colony numbers in A549 and H1299 cells. (E) Cell proliferation was also determined by EdU staining in A549 and H1299 cells transfected with circ_0030998 plasmid or vector. Magnification, ×100; scale bar = 100 μm. (F,G) After transfection with the circ_0030998 plasmid, the representative results of the Transwell assay show changes in the migration and invasion capacities of A549 and H1299 cells (scale bar, 200 μm). Data represent the mean ± SD from three independent experiments. Student’s t‐test with two biological dependent or independent replicates was used to determine statistical significance; **P < 0.01

To further examine the biological functions of circ_0030998 in lung cancer, we successfully constructed the circ_0030998‐overexpressing plasmid. After transfection with the circ_0030998‐overexpressing plasmid in A549 and H1299 cells, the transfection effect was confirmed through qRT–PCR analysis (Fig. 2A, *P* < 0.001). Next, a series of functional experiments were conducted. First, the CCK‐8 assay revealed that cell proliferation was significantly lower in the circ_0030998‐overexpressing group than in the vector group (*P* < 0.01, Fig. 2B,C). Similarly, the colony formation and EdU staining assays also revealed that the overexpression of circ_0030998 notably suppressed the proliferation of A549 and H1299 cells (*P* < 0.01, Fig. 2D,E). Second, the Transwell results demonstrated that the overexpression of circ_0030998 blocked cell migration and invasion progression in A549 and H1299 cells (*P* < 0.01, Fig. 2F,G). In summary, we demonstrated that the overexpression of circ_0030998 significantly inhibited the proliferation, migration, and invasion of lung cancer cells.

### 2.3 Knockdown of circ_0030998 promoted the malignant behavior of lung cancer cells

**Fig. 3:**
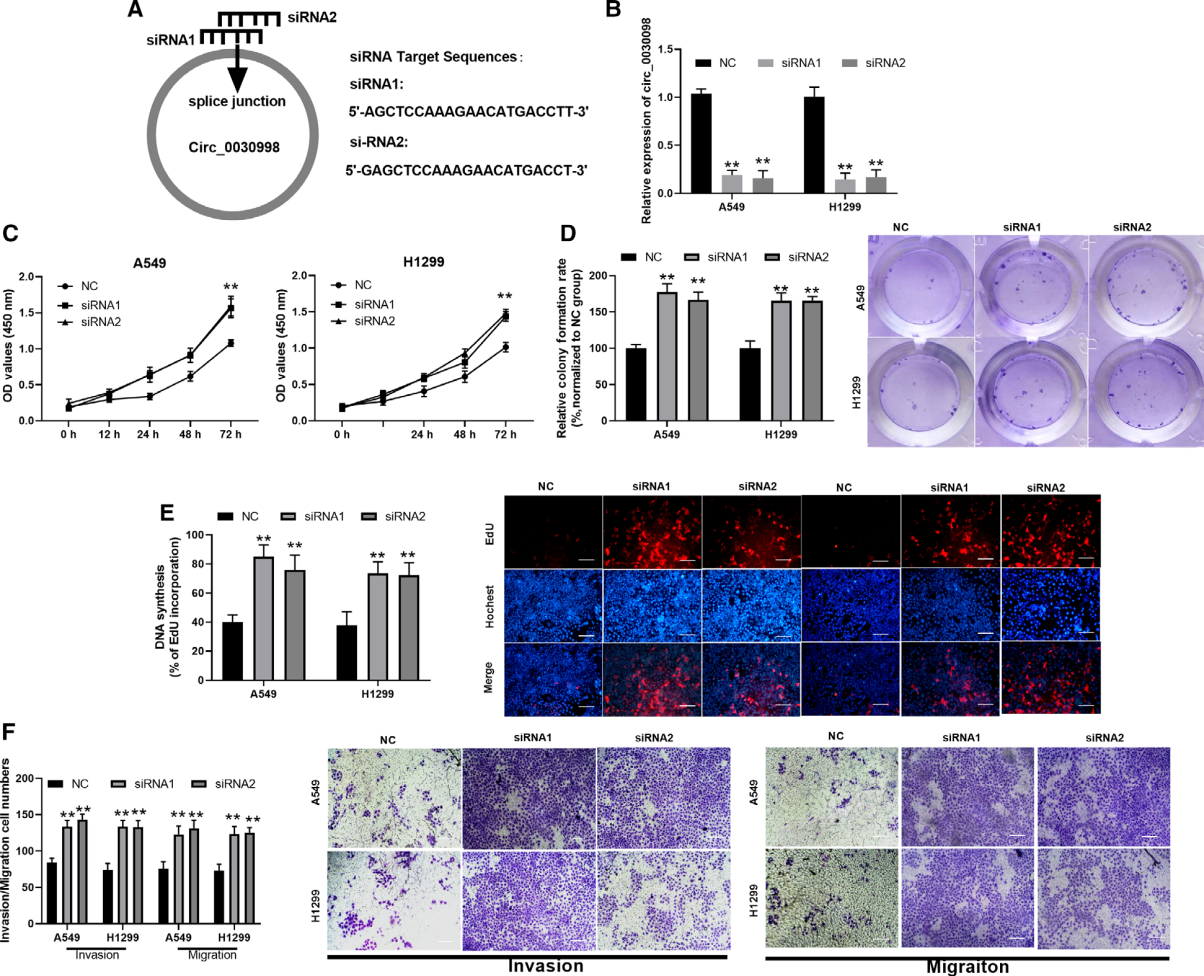
Knockdown of circ_0030998 promoted the malignant behavior of lung cancer cells. (A) In accordance with the splice junction of circ_0030998, the sequences of circ_0030998 siRNAs were designed. (B) The silencing effect of circ_0030998 was also identified using qRT–PCR assay in A549 and H1299 cells after transfection with circ_0030998 siRNAs. The promoting effect of circ_0030998 knockdown by siRNAs on cell proliferation was identified through CCK‐8 (C), colony formation (D), and EdU staining assays (E) in A549 and H1299 cells. (F) Transwell assay was adopted to determine the changes in the abilities of cells to migrate and invade in si‐circ_0030998#1‐ or si‐circ_0030998#2‐transfected A549 and H1299 cells (scale bar, 200 μm). Data represent the mean ± SD from three independent experiments. Student’s t‐test with two biological dependent or independent replicates was used to determine statistical significance;**P < 0.01

Next, we further investigated the influences of circ_0030998 knockdown on the malignant progression of lung cancer cells by assessing proliferation, migration, and invasion. In accordance with the splice junction of circ_0030998, we designed circ_0030998 siRNAs, and the target sequences of siRNAs were exhibited (Fig. 3A). qRT–PCR analysis was then conducted to identify the transfection effects of circ_0030998 siRNAs, and the results indicated that the silencing efficacy of circ_0030998 was remarkable in A549 and H1299 cells (*P* < 0.01, Fig. 3B). Functionally, the data from the CCK‐8 assay illustrated that the proliferation of A549 and H1299 cells was markedly elevated in the circ_0030998‐silenced group compared with that in the NC group (*P* < 0.01, Fig. 3C). At the same time, the results of colony formation and EdU staining assays showed that the proliferation capacities of circ_0030998‐silenced A549 and H1299 cells were notably higher than those of the NC group (*P* < 0.01, Fig. 3D,E). More importantly, we also revealed that knockdown of circ_0030998 observably accelerated the migration and invasion of A549 and H1299 cells (*P* < 0.01, Fig. 3F). Consequently, we further verified that silencing circ_0030998 markedly facilitated the progression of lung cancer.

### 2.4 hsa_circ_0030998 reduced Taxol resistance in Taxol‐resistant lung cancer cells

**Fig. 4:**
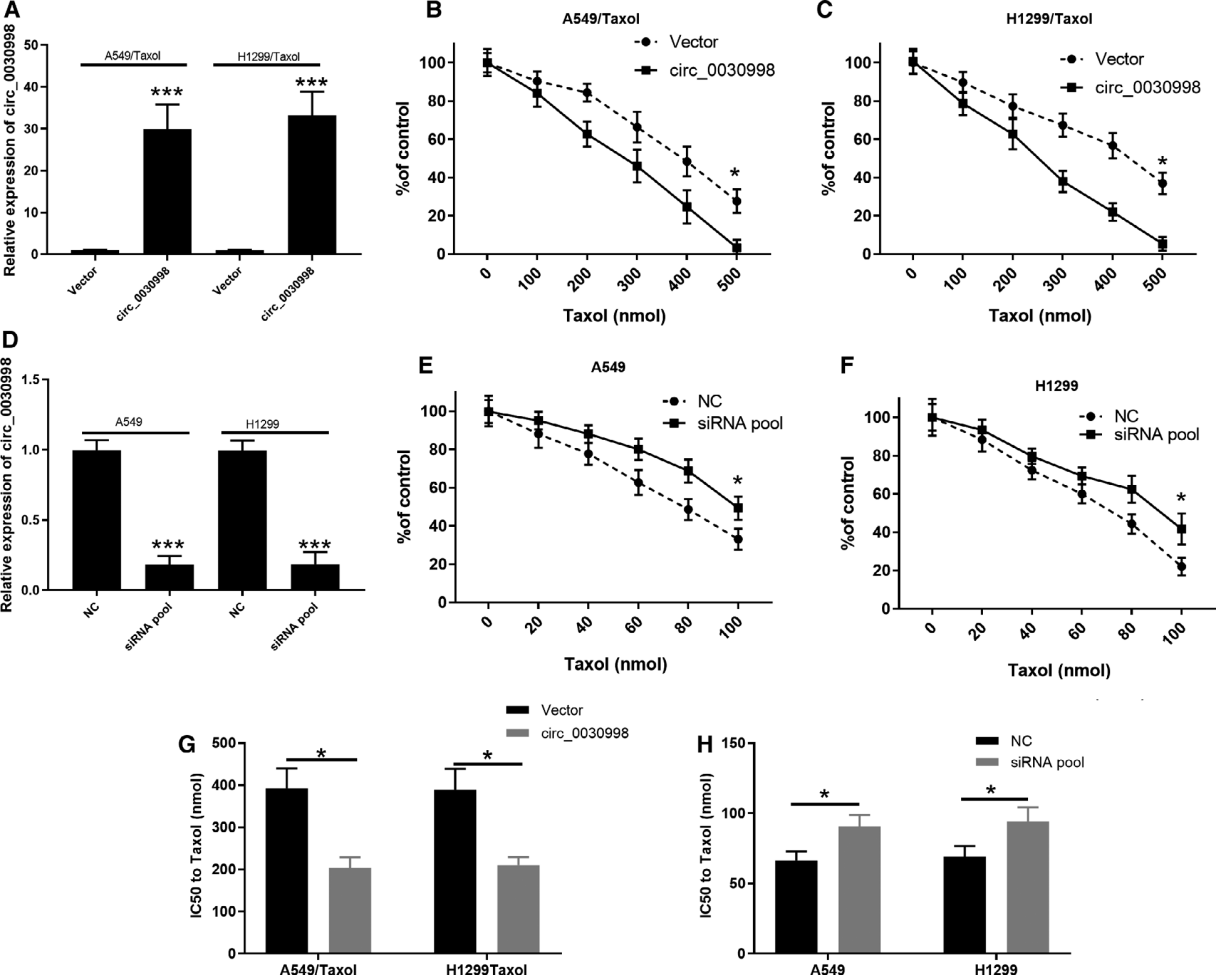
hsa_circ_0030998 reduced Taxol resistance in Taxol‐resistant lung cancer cells. (A) qRT–PCR confirmed the overexpression of circ_0030998 in Taxol‐resistant A549 and H1299 cells. (B,C) CCK‐8 assay was applied to determine the influences of different concentrations of Taxol on the inhibition ratio of Taxol‐resistant A549 and H1299 cells after circ_0030998 overexpression. (D) Knockdown of circ_0030998 by siRNAs was identified through qRT–PCR assay in Taxol‐resistant A549 and H1299 cells. (E,F) After treatment with different concentrations of Taxol, the inhibition ratio of Taxol‐resistant A549 and H1299 cells was assessed by CCK‐8 assay after circ_0030998 knockdown. (G,H) The IC50 of Taxol was confirmed through CCK‐8 assay in Taxol‐resistant A549 and H1299 cells after circ_0030998 overexpression or knockdown. Data represent the mean ± SD from three independent experiments. Student’s t‐test with two biological dependent or independent replicates was used to determine statistical significance;*P < 0.05, ***P < 0.001

To verify the impacts of circ_0030998 overexpression and knockdown on the resistance and cytotoxicity of Taxol‐resistant lung cancer cells, circ_0030998 expression was detected first in circ_0030998‐overexpressing Taxol‐resistant and circ_0030998‐silenced lung cancer cells. As demonstrated in Fig. 4A, circ_0030998 expression was significantly increased in circ_0030998‐overexpressing A549/Taxol and H1299/Taxol cells compared with that in vector‐transfected cells (*P* < 0.001). CCK‐8 results revealed that Taxol treatment could dramatically reduce the cell viability in circ_0030998‐overexpressing plasmid‐ or vector‐transfected A549/Taxol and H1299/Taxol cells, especially in the circ_0030998 overexpression group (*P* < 0.05, Fig. 4B,C). Moreover, we examined the level of circ_0030998 in circ_0030998‐silenced A549 and H1299 cells, and our results revealed that circ_0030998 was prominently downregulated in circ_0030998‐silenced A549 and H1299 cells versus that in NC cells (*P* < 0.001, Fig. 4D). We also discovered that the cell viability was gradually reduced with increasing Taxol concentration in circ_0030998‐silenced or NC‐transfected A549 and H1299 cells, while the degree of reduction in cell viability was lower in the circ_0030998 siRNA group than in the NC group (*P* < 0.05, Fig. 4E,F). We also demonstrated that the IC50 of Taxol in A549/Taxol and H1299/Taxol cells was dramatically decreased in the circ_0030998 overexpression group compared with the vector transfection group (*P* < 0.05, Fig. 4G). The IC50 of Taxol in A549 and H1299 cells was significantly increased in the circ_0030998 knockdown group compared with the NC transfection group (*P* < 0.05, Fig. 4H). As a result, we also confirmed that hsa_circ_0030998 could enhance Taxol sensitivity in Taxol‐resistant lung cancer cells.

### 2.5 circ_0030998 functioned as a miR‐558 sponge

**Fig. 5:**
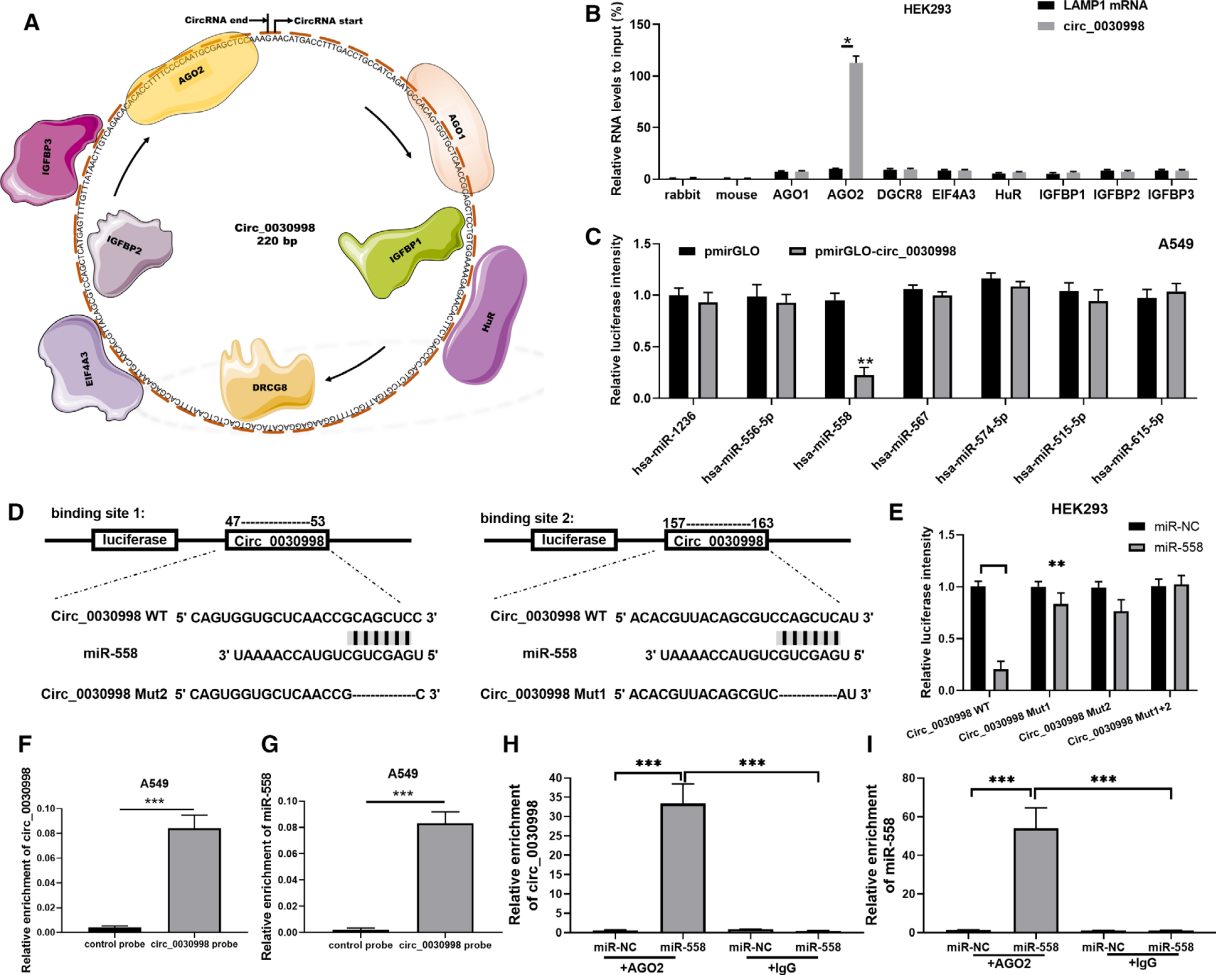
circ_0030998 functioned as a miR‐558 sponge. (A) The circular interactome was utilized to predict the RBPs that may interact with circ_0030998, and eight proteins were predicted and exhibited. (B) A pull‐down assay was adopted to verify the interaction between circ_0030998 and the 8 proteins. (C) The miRNAs that may bind to circ_0030998 were also predicted through the circular interactome, and the regulatory relationships were confirmed by luciferase reporter assay. (D) Putative miR‐558 binding sequences in circ_0030998 are displayed, and reporter gene plasmids were constructed. (E) The luciferase intensity between circ_0030998 and miR‐558 was assessed through a dual‐luciferase reporter gene assay. (F,G) The enrichments of circ_0030998 and miR‐558 were also verified by RNA pull‐down assay in A549 cells. (H,I) Ago2‐IP assay was then applied to confirm the enrichments of circ_0030998 and miR‐558 in A549 cells. Data represent the mean ± SD from three independent experiments. Student’s t‐test with two biological dependent or independent replicates was used to determine statistical significance; *P < 0.05, **P < 0.01, ***P < 0.001

Subsequently, we further investigated the possible regulatory proteins and miRNAs of circ_0030998. First, the results of our qRT–PCR analysis showed that circ‐0030998 was located in the cytoplasm of A549 and H1299 cells (Fig. S3). Therefore, we speculated that the mechanisms of miRNA sponges or RNA‐binding protein (RBP) sponges might be applied to participate in the functional regulation of lung cancer cells. Moreover, we adopted a circular interactome to predict the RBPs that might interact with circ_0030998, and we discovered 8 potential RBPs. As exhibited in Fig. 5A, the 8 potential RBPs were AGO2, IGFBP3, IGFBP2, EIF4A3, DRCG8, HuR, IGFBP1, and AGO1. Next, the interactions between circ_0030998 and the 8 proteins were verified through the application of a pull‐down assay. The results revealed that the relative level of circ_0030998 was significantly increased only in the anti‐AGO2 group, suggesting that AGO2 interacted with circ_0030998 (*P* < 0.05, Fig. 5B). In addition, bioinformatic analysis was conducted to predict the underlying miRNAs of circ‐0030998, and 6 miRNAs were confirmed in A549 cells, including miR‐1236, miR‐556‐5p, miR‐558, miR‐567, miR‐574‐5p, miR‐515‐5p, and miR‐615‐5p. We also discovered that only the relative luciferase intensity of miR‐558 was significantly decreased in the pmirGLO‐circ‐0030998 group (*P* < 0.01, Fig. 5C). We further tested the regulation of miR‐558 by circ‐0030998 through a luciferase reporter assay. The putative two binding sites (47–53 and 157–163) of circ_0030998 were displayed, and the corresponding wild‐type and mutant circ_0030998 plasmids were also constructed (Fig. 5D). Our results revealed that the luciferase intensity was notably attenuated in HEK293 cells cotransfected with wild‐type circ_0030998 and miR‐558 mimics (*P* < 0.01, Fig. 5E). Moreover, the results of the RNA pull‐down assay also confirmed that the enrichments of circ_0030998 and miR‐558 were dramatically enhanced in A549 cells (*P* < 0.001, Fig. 5F,G). The data from the Ago2‐IP assay also verified that after anti‐AGO2 treatment, the relative enrichment of circ_0030998 or miR‐558 was prominently strengthened in the miR‐558 group compared with the miR‐NC group (*P* < 0.001, Fig. 5H,I). Consequently, these results revealed that circ_0030998 may serve as a miR‐558 sponge.

### 2.6 hsa_circ_0030998 attenuated the Taxol resistance, proliferation, migration, and invasion of lung cancer cells by inhibiting miR‐558

**Fig. 6:**
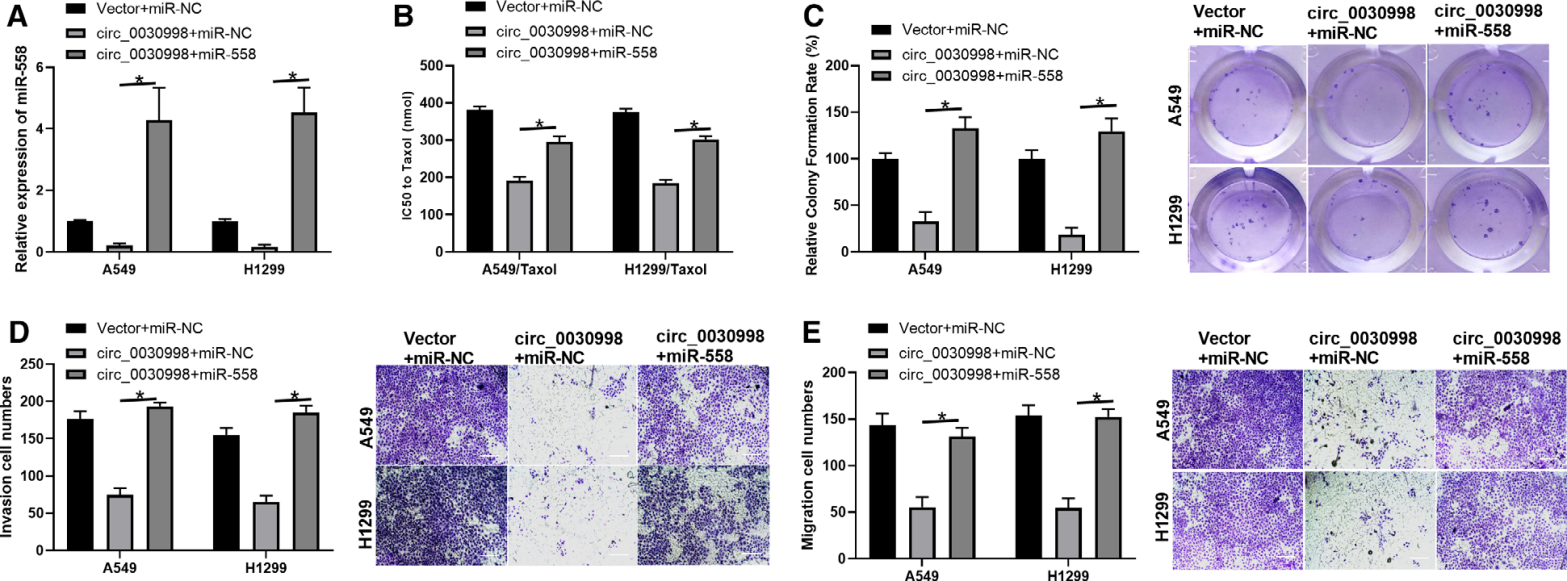
hsa_circ_0030998 attenuated the Taxol resistance, proliferation, migration, and invasion of lung cancer cells by inducing the inhibition of miR‐558. Taxol‐resistant or Taxol‐sensitive A549 and H1299 cells were cotransfected with circ_0030998 plasmid and miR‐558 mimics. (A) qRT–PCR analysis of miR‐558 expression. (B) The IC50 of Taxol was examined by CCK‐8 assay. (C) Cell proliferation was monitored through the application of colony formation assay. (D,E) Transwell assays revealed changes in the migration and invasion capacities of transfected A549 and H1299 cells (scale bar, 200 μm). Data represent the mean ± SD from three independent experiments. Student’s t‐test with two biological dependent or independent replicates was used to determine statistical significance; *P < 0.05

Next, rescue assays were performed to confirm the hsa_circ_0030998/miR‐558 axis in lung cancer cell tumorigenesis. qRT–PCR analysis showed that the overexpression of circ_0030998 significantly reduced the level of miR‐558, while miR‐558 attenuated this reduction in A549 and H1299 cells (*P* < 0.05, Fig. 6A). The overexpression of circ_0030998 observably decreased the IC50 of Taxol, while this decrease was reversed by miR‐558 mimics in A549/Taxol and H1299/Taxol cells (*P* < 0.05, Fig. 6B). We also demonstrated that the overexpression of circ_0030998 notably lowered the relative colony formation rate, which was also markedly reversed by miR‐558 mimics in A549 and H1299 cells (*P* < 0.05, Fig. 6C). Moreover, our data revealed that the upregulation of circ_0030998 prominently decreased the migration and invasion capacities of A549 and H1299 cells and that these inhibitory effects could be abolished by miR‐558 mimics (*P* < 0.05, Fig. 6D,E). Overall, we indicated that circ_0030998 suppresses lung cancer progression and Taxol resistance by regulating miR‐558.

### 2.7 miR‐558 markedly downregulated MMP17 and MMP1 expression through targeted regulation

**Fig. 7:**
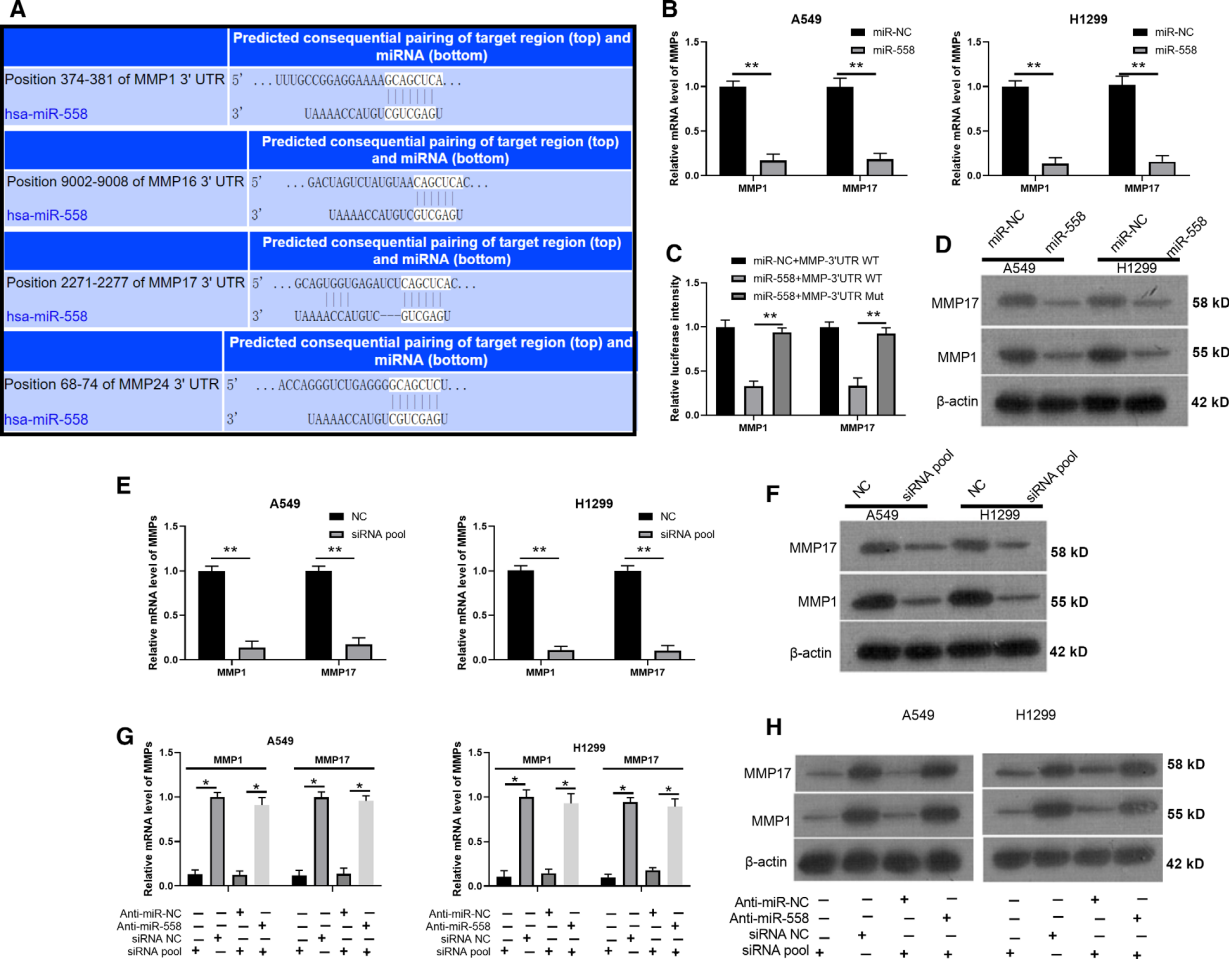
miR‐558 markedly downregulated MMP17 and MMP1 expression through targeted regulation. (A) The targeted genes of miR‐558 were predicted through the TargetScan database, and the binding sites between miR‐558 and MMP1, MMP16, MMP17, and MMP24 were exhibited. (B) MMP1 and MMP17 expression was monitored by qRT–PCR in A549 and H1299 cells transfected with miR‐558 mimics. (C) Wild‐type and mutant MMP1 and MMP17 plasmids for miR‐558 were constructed, and the interaction was confirmed via luciferase reporter assay in 293T cells. (D) The effects of miR‐558 on the expression of MMP1 and MMP17 were identified by western blot assay in A549 and H1299 cells. (E,F) MMP1 and MMP17 expression was determined via qRT–PCR and western blot assays in A549 and H1299 cells transfected with siRNA pool. (G,H) qRT–PCR and western blotting analyses were carried out in A549 and H1299 cells cotransfected with anti‐miR‐558 and MMP1 or MMP17 siRNAs. Data represent the mean ± SD from three independent experiments. Student’s t‐test with two biological dependent or independent replicates was used to determine statistical significance; *P < 0.05, **P < 0.01

Furthermore, our prediction method indicated high binding scores between miR‐558 and MMP1, MMP16, MMP17, or MMP24 3ʹ‐UTRs, and the binding sites between miR‐558 and MMP1, MMP16, MMP17, and MMP24 are shown in Fig. 7A. We confirmed that miR‐558 markedly downregulated MMP1 and MMP17 in A549 and H1299 cells (*P* < 0.001, Fig. 7B). Subsequently, the data of the dual‐luciferase reporter assay also confirmed that the luciferase intensity driven by WT‐MMP1 or WT‐MMP17 was notably weakened by transfection with miR‐558 mimics (*P* < 0.01, Fig. 7C). In addition, we proved that miR‐558 observably downregulated the protein levels of MMP1 and MMP17 in A549 and H1299 cells (Fig. 7D). Next, MMP1 and MMP17 expression levels were verified after transfection with a pool of siRNAs; our results also showed that MMP1 and MMP17 were significantly silenced through knockdown using the respective siRNAs in A549 and H1299 cells (*P* < 0.01, Fig. 7E,F). More importantly, we also performed rescue experiments to reveal the impacts of miR‐558 on MMP1 and MMP17 expression. Our results suggest that anti‐miR‐558 markedly reverses the downregulation of MMP1 and MMP17 expression, which is mediated by the respective siRNAs in A549 and H1299 cells (*P* < 0.01, Fig. 7G,H). As a result, we demonstrated that MMP17 and MMP1 are the target genes of miR‐558 and that they can be downregulated by miR‐558 in A549 and H1299 cells.

## 3 Discussion

As part of the noncoding RNA family, circRNAs have been extensively studied in recent years [21]. Similar to lncRNAs and miRNAs, circRNAs can interact with RNA‐binding proteins (RBPs) and even translate them directly into proteins [22]. Due to their stability, conservatism, and spatiotemporal specificity, circRNAs have unique advantages in tumor diagnosis and treatment [17]. An increasing number of lung cancer‐associated circRNAs have been disclosed, such as hsa_circ_100395 [23], hsa_circRNA_103809 [24], circNOL10 [25], hsa_circ_0008305 [26], hsa_circ_0001946 [27], and hsa_circ_0020123 [28]. In this study, we surveyed the related effect and mechanism of a novel hsa_circ_0030998 in lung cancer. We discovered that circ_0030998 is encoded by exon 3 of LAMP1 mRNA and that circ_0030998 is more stable than linear LAMP1. Moreover, we verified that circ_0030998 is downregulated in lung cancer tissues and cells, as well as Taxol‐resistant A549 and H1299 cells. Functionally, we also proved that the overexpression of circ_0030998 could markedly inhibit the proliferation, migration, and invasion of lung cancer cells, and knockdown of circ_0030998 notably accelerates the proliferation, migration, and invasion of lung cancer cells. Moreover, we also verified that circ_0030998 attenuates Taxol resistance in Taxol‐resistant lung cancer cells.

Studies have confirmed that the functions of circRNAs are usually relevant to their different localization in cells [21, 29]. circRNAs in the cytoplasm usually function by binding to miRNAs or proteins, and some circRNAs in the cytoplasm also have protein‐coding functions [30]. circRNAs in the nucleus usually can bind to RNA polymerase II and play a role in transcriptional regulation in the nucleus [31]. In the present study, we revealed that circ‐0030998 is located in the cytoplasm of A549 and H1299 cells and indicated that circ_0030998 serves as a miR‐558 sponge. In addition, we demonstrated that miR‐558 reverses the inhibitory effects of hsa_circ_0030998 on the Taxol resistance, proliferation, migration, and invasion of lung cancer cells. The circ_0030998/miR‐558 axis is a new signaling pathway that may serve as a therapeutic target in lung cancer tumorigenesis.

miR‐558 has been shown to promote a variety of processes in cancers, such as neuroblastoma [32], gastric cancer [33], and bladder cancer [34]. miR‐558 has also been confirmed to be involved in the invasion and migration of trophoblasts by targeting TIMP4 [35]. In our study, we found that miR‐558 has binding sites for MMP1, MMP16, MMP17, and MMP24 3'‐UTRs, and only MMP17 and MMP1 were demonstrated to be significantly downregulated by miR‐558 through targeted regulation in lung cancer cells. Matrix metalloproteinases (MMPs) can degrade extracellular matrix (ECM) and collagen and significantly contribute to tumor progression, especially invasion, metastasis, and angiogenesis [36]. In the present study, we identified two important MMPs, MMP1 and MMP17, that are the direct target genes of miR‐558 in lung cancer cells. Collectively, circ_0030998 is capable of inhibiting lung cancer cell invasion and migration, which may be mediated by inhibition of miR‐558‐dependent downregulation of MMPs.

## 4 Conclusion

Our study verified that hsa_circ_0030998 can inhibit proliferation, migration, invasion, and Taxol resistance by sponging miR‐558. Therefore, the hsa_circ_0030998/miR‐558 axis might be a possible diagnostic and therapeutic target in lung cancer patients. In future studies, the hsa_circ_0030998/miR‐558/MMP1/MMP17 pathway will be further confirmed and clarified through a series of *in vivo* experiments and through studies in more lung cancer cells.

## 5 Materials and Methods

### 5.1 Clinical samples

Forty‐six pairs of surgically excised lung cancer and adjacent tissues (approximately 5 cm away from cancer tissue) were collected from Tianjin First Central Hospital. Lung cancer was verified through pathological measurement. After excision, the tissues were immediately stored in liquid nitrogen. Among the patients, 28 were male and 18 were female, aged 39–74 years; 24 cases of well/moderate differentiation and 22 cases of poor differentiation; 35 cases of TNM stages I‐II; and 11 cases of TNM stages III–IV were included. Before collection, we obtained informed consent from the patients. Our study was also approved by the Ethics Committee of the Tianjin First Central Hospital. The study methodologies confirmed to the standards set by the Declaration of Helsinki.

### 5.2 Cell culture and treatment

Human bronchial epithelial (HBE) cells, 4 lung cancer cell lines (A549, H1299, H358, and PC9), and HEK293 cells were purchased from ATCC. HBE cells were grown in DMEM (HyClone, Cat. No. SH30022.01B, Logan, UT, USA), and A549, H1299, H358, PC9, and HEK293 cells were cultured in RPMI 1640 medium RPMI 1640 (Gibco; Cat No. 31800, Grand Island, NY, USA). Both culture media included 10% fetal bovine serum (FBS, HyClone, Cat. No. SH30087.01), and all cells were incubated at 37 °C in 5% CO_2_. Taxol‐resistant A549 and H1299 cells were also established by increasing the concentration gradient method.

### 5.3 Cell transfection

The hsa_circ_0030998‐overexpressing plasmid and the corresponding vector were successfully constructed by Nanjing Dehengwen Biological Technology Co., Ltd. (Nanjing, China). The sequences of circ_0030998 siRNAs were designed, and the target sequences were circ_0030998 siRNA1: 5’‐AGCTCCAAAGAACATGACCTT‐3’; and circ_0030998 siRNA2: 5’‐GAGCTCCAAAGAACATGACCT‐3’. circ_0030998 siRNAs, MMP1 siRNAs, MMP17 siRNAs, negative control (NC), miR‐1236 mimics, miR‐556‐5p mimics, miR‐558 mimics, miR‐567 mimics, miR‐574‐5p mimics, miR‐515‐5p mimics, miR‐615‐5p mimics, and miR‐NC were synthesized by GenePharma (Shanghai, China). A549 and H1299 cells were evenly seeded into 6‐well plates at a density of 1 × 10^5^ cells/well. After incubation for 8 h, cells were transfected with the specified plasmids, miRNA mimics, and siRNAs by applying Lipofectamine 3000 Reagent (Invitrogen, Waltham, MA, USA) in line with the experimental instructions. The detailed cell phenotype assays, including CCK‐8, colony formation, EdU staining, wound healing, and Transwell assays, are described in the Supporting Information.

### 5.4 CCK‐8 assay

The suspension of the transfected A549 and H1299 cells was transferred to 96‐well plate, and the addition amount of cells in each well was 100 μL and 2 × 10^3^ cells. After being incubated in an incubator for 0, 12, 24, 48, and 72 h, 10 μL CCK‐8 was added to each well and incubated in a cell incubator for 3 h. The absorbance was determined through a using Infinite® 200 PRO (FPRO‐T; Tecan, Seestrasse, Switzerland) at 450 nm.

### 5.5 Colony formation assay

A549 and H1299 cells (1 × 10^3^ cells/well) in each group were plated into the 6‐well plates and cultured with complete medium which were changed every 3 days. After 2 weeks, cells were fixed and stained with 0.1% crystal violet (Sigma‐Aldrich, St. Louis, MO, USA). The cell clones were observed under a microscope.

### 5.6 EdU staining

A549 and H1299 cells in each group were hatched in complete medium with 50 μmol L^−1^ EdU solution (Solarbio, Cat. No. CA1170, Beijing, China) for 2 h. After fixing, cells were decolored using 50 μL glycine (2 mg mL^−1^), and the cells were observed using a fluorescence microscope.

### 5.7 Wound healing assay

The transfected A549 and H1299 cells were evenly spread in 6‐well plate, the scratch operation was performed when the cell density reached 100%. The bottom of 6‐well plate was evenly and vertically drawn as a straight line using the sterilized yellow head. After washing with PBS, cells were cultured in the complete medium. Scratches were recorded at 0 and 24 h.

### 5.8 Transwell assay

A549 and H1299 cells in each group were adjusted to 1 × 10^5^ cells ml^−1^ using serum‐free medium, and 100 μL cells were added into the upper layer of Transwell chamber, and 600 μL medium including 10% FBS was added into lower layer of Transwell chamber. After 24 h of incubation at 37 °C, cells were fixed with 70% methanol. After the cells in the upper compartment were removed, the cells were stained with 4% crystal violet (Sigma‐Aldrich). The migrating cells were observed and recorded under a microscope. For cell invasion, Matrigel was diluted in 1 ∶ 3 ratio with serum‐free medium, and 40 μL diluted Matrigel was spread on the upper layer of Transwell chamber. The other steps were the same as the migration experiment.

### 5.9 Polymerase chain reaction and qRT–PCR assays

TRIzol reagent (Invitrogen) was applied to extract the total RNA from the treated cells and tissues in each group. Then, reverse transcription was conducted using the First‐Strand cDNA Synthesis Kit (Takara, Shiga, Japan), and the reaction conditions were 42 °C, 30 min and 85 °C, 5 s. Genes were examined by real‐time PCR assay through the application of SYBR Green qPCR Super Mix (Invitrogen) on the ABI Prism® 7500 Sequence Detection System. PCR assays were also conducted to analyze circ_0030998 and LAMP1 expression via agarose gel electrophoresis, and the results were obtained using a UV gel imaging system (Wealtec, Sparks, NV, USA). The sequences of all primers are presented in Table 2.

**Table 2:** The sequences of primers in qRT–PCR assay

ID	Sequence (5’‐ 3’)
LAMP1	Forward: AGCTTTAAAGGCCAGGACGG
LAMP1	Reverse: TGTACACAGCGCAGAACAGG
circ_0030998	Forward: GAAGGTCACTCGTGGTGAGG
circ_0030998	Reverse: CTGGTCCCGTGTACAATCCC
MMP1	Forward: AGCTAGCTCAGGATGACATTGATG
MMP1	Reverse: CTCCCCGAATCGTAGTTATAGCAT
MMP17	Forward: AGGAGCTGTCTAAGGCCATC
MMP17	Reverse: CTCCACGACAGGTTCCTCTT
GAPDH	Forward: TGTTCGTCATGGGTGTGAAC
GAPDH	Reverse: ATGGCATGGACTGTGGTCAT
miR‐558	RiboBio
miR‐558	RiboBio
U6	Forward: CTCGCTTCGGCAGCACATATACT
U6	Reverse: ACGCTTCACGAATTTGCGTGTC

### 5.10 Luciferase reporter gene assay

WT‐pmirGLO⁃hsa_circ_0030998 and Mut‐pmirGLO⁃hsa_circ_0030998 were designed and built by Wuhan Hualian Biotechnology Co., Ltd (Wuhan, China). HEK293T cells (1 × 10^5^ cells/well) were plated in a 6‐well plate and cultured for 8 h. The cells were then cotransfected with the plasmids or miR‐1236 mimics, miR‐556‐5p mimics, miR‐558 mimics, miR‐567, miR‐574‐5p mimics, miR‐515‐5p mimics, or miR‐615‐5p mimics using Lipofectamine 3000 Reagent (Invitrogen). After 48 h, all cells were harvested and examined by applying the Dual‐Luciferase Reporter Assay System (Promega, Madison, WI, USA).

### 5.11 Western blotting analysis

A549 and H1299 cells in each group were collected and washed with precooled PBS, and then, cells were treated with ice‐cold RIPA (Solarbio) with protease inhibitors. After centrifugation, the supernatant was extracted, and the protein concentration was examined by applying the Bradford Protein Assay Kit (Beyotime, China). Then, the protein in each group was separated through SDS/PAGE electrophoresis and transferred to a PVDF membrane (Thermo Fisher Scientific, Waltham, MA, USA). After blocking, the membranes were covered with primary antibody overnight at 4 °C and then secondary antibody for 2 h. After treatment with the ECL Substrate Kit (Thermo Scientific), the protein bands were displayed using an iBright FL1500 Imaging System (Thermo Fisher Scientific).

### 5.12 RNA pull‐down assay

The cleaved A549 cells were treated with biotin (Bio)‐labeled oligonucleotide probes against circ_0030998 at 25 °C. After 2 h, streptavidin‐coupled Dynabeads (Invitrogen) were applied to obtain circ_0030998/miR‐558 complexes. circ_0030998/miR‐558/bead complexes were then treated with RIP buffer (Millipore, Burlington, MA, USA) supplemented with proteinase K. The results were then monitored through qRT–PCR analysis.

### 5.13 Ago2‐Immunoprecipitation assay

hsa_circ_0030998 or miR‐558‐Ago2 was adopted to treat the cleaved A549 cells. After 48 h, the proteins were isolated and incubated with 2 μg of the corresponding antibody for 4 h at 4 °C. After washing, qRT–PCR assays were conducted for circ_0030998 and miR‐558.

### 5.14 Statistical analysis

The measurement data of the research results are presented as the mean ± SD, and the results were processed using SPSS software (ver. 20.0, SPSS, Inc., Chicago, IL, USA). Analysis of variance (ANOVA) was applied for comparisons between multiple groups, and Student's *t*‐test was utilized for comparisons between two groups. The data of each group were significantly different when *P* < 0.05.

## Conflict of interest

The authors declare no conflicts of interest.

## Author contributions

XL and WZ conceived, designed, and analyzed experiments; XL, YF, BY, TX, and HR performed experiments and wrote the manuscript; XY, LL, ML, and XL helped in conceiving and/or analyzing the experiments and provided reagents.

## Supporting information


**Fig S1.** The stability identification of hsa_circ_0030998 in lung cancer cells.
**Fig S2.** Hsa_circ_0030998 was derived from LAMP1 mRNA.
**Fig S3.** Circ‐0030998 was located in the cytoplasm.
