# Supplementary material for: A novel circular RNA, hsa_circ_0030998 suppresses lung cancer tumorigenesis and Taxol resistance by sponging miR‐558
Source: Mol Oncol. 2021 Feb 10;15(8):2235–48. doi: 10.1002/1878-0261.12852 (PMC8333779; doi:10.1002/1878-0261.12852)
Supplement: Supplementary file 1 — Fig S1. The stability identification of hsa_circ_0030998 in lung cancer cells. Fig S2. Hsa_circ_0030998 was derived from LAMP1 mRNA. Fig S3. Circ‐0030998 was located in the cytoplasm. [file MOL2-15-2235-s001.docx]

**Supplementary Figures**


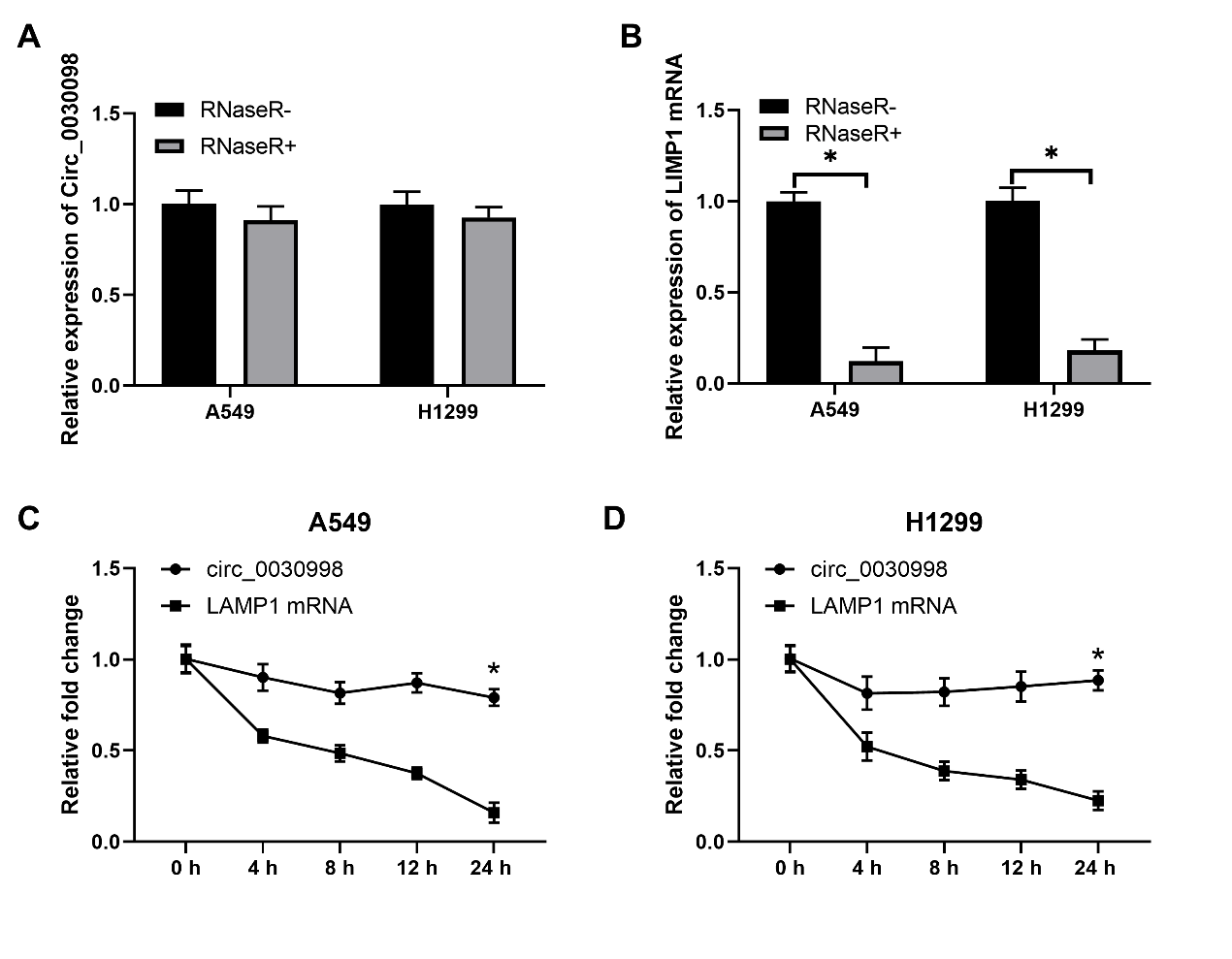


**Figure S1**. The stability identification of **hsa_****circ_0030998 in lung cancer cells.** **(A-B)** The expressions of hsa_circ_0030998 and LAMP1 were identified by qRT-PCR assay in A549 and H1299 cells after treatment with RNaseR. **(C-D)** The resistance of hsa_circ_0030998 and LAMP1 to actinomycin were determined through qRT-PCR analysis in A549 and H1299 cells. Data represent the mean±SD from three independent experiments. Student’s t-test with two biological dependent or independent replicates was used to determine statistical significance; **P* < 0.05.
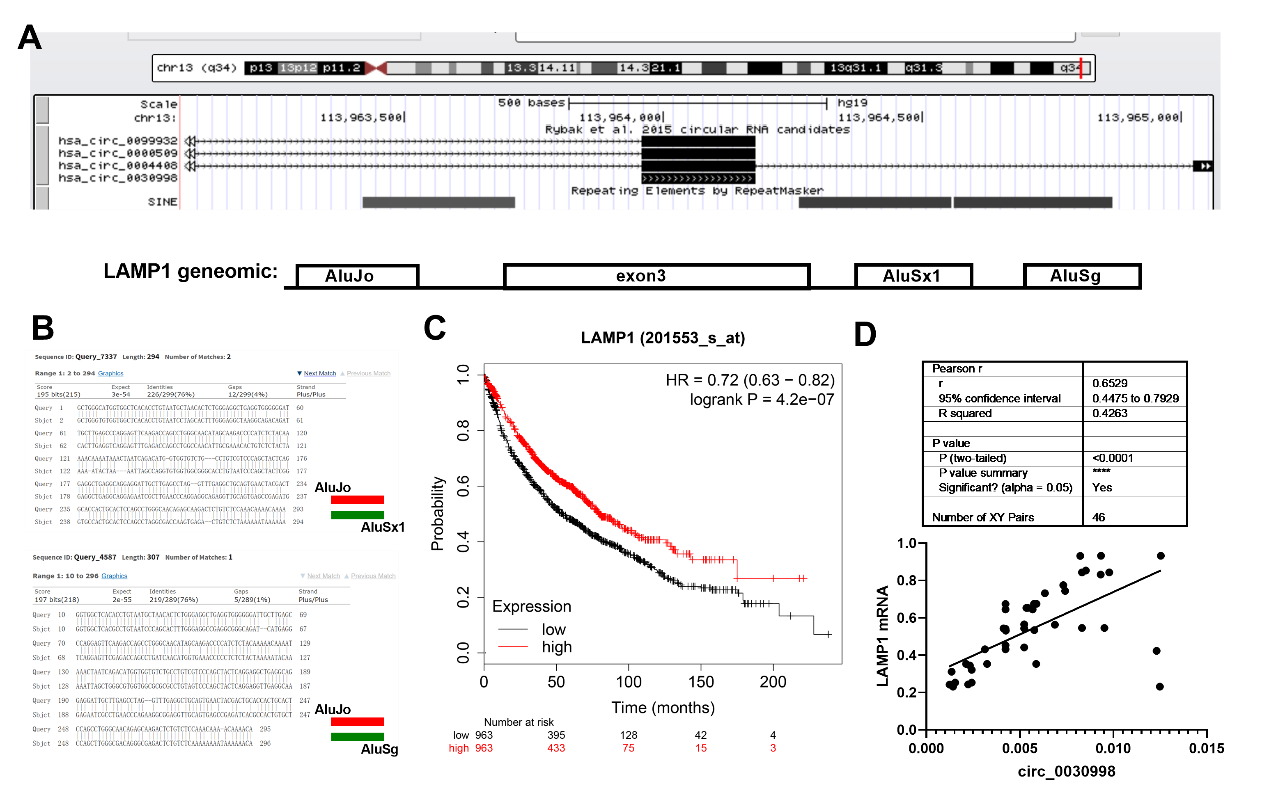


**Figure S2**. **Hsa_circ_0030998 was derived from LAMP1 mRNA.** **(A)** The position of hsa_circ_0030998 was exhibited on the chromosome. **(B)** The sequence alignment of hsa_circ_0030998 was displayed. **(C)** The prognosis of LAMP1 was analyzed through Kaplan-Meier Plotter. **(D)** Correlation analysis between hsa_circ_0030998 and LAMP1 in lung cancer (r=0.6529, *P*<0.0001).


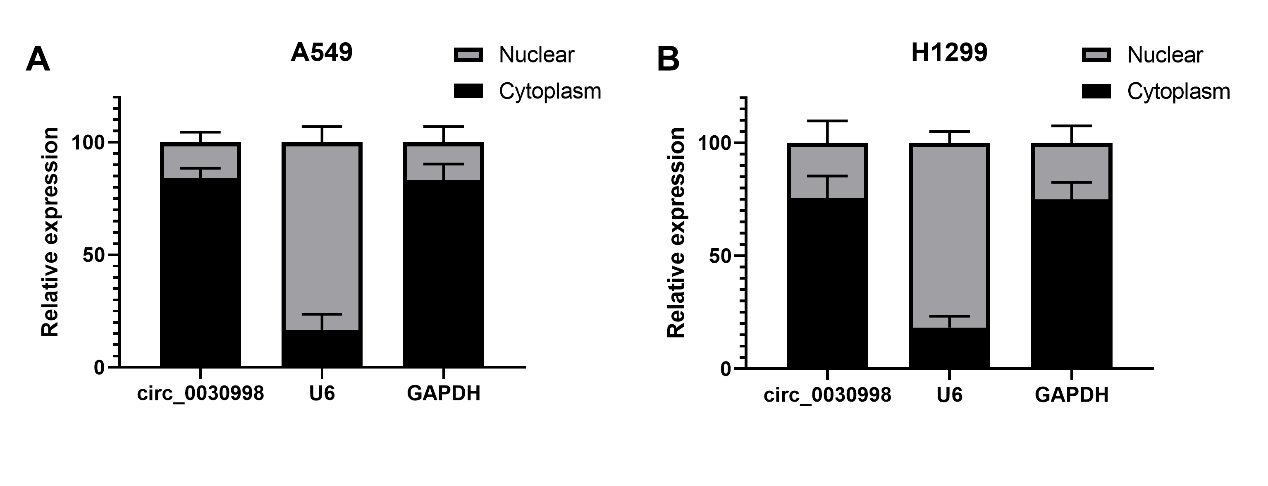


**Figure S3.** **Circ-0030998 was located in the** **cytoplasm. (A-B)** Circ-0030998 expression was confirmed through qRT‐PCR analysis in the cytoplasm and nuclear. Data represent the mean±SD from three independent experiments.
